# Exploring Nociceptive–Analgesic Balance and EEG Modulation Patterns During General Anesthesia Using Holo‐Hilbert Spectral Analysis

**DOI:** 10.1155/prm/5504074

**Published:** 2026-02-03

**Authors:** Chun-Ning Ho, Norden E. Huang, Jen-Yin Chen, Albert C. Yang

**Affiliations:** ^1^ Institute of Brain Science, National Yang Ming Chiao Tung University, Taipei, Taiwan, nctu.edu.tw; ^2^ School of Medicine, College of Medicine, National Sun Yat-Sen University, Kaohsiung City, Taiwan, nsysu.edu.tw; ^3^ Department of Anesthesiology, Chi Mei Medical Center, Tainan, Taiwan, chimei.org.tw; ^4^ Digital Medicine and Smart Healthcare Research Center, National Yang Ming Chiao Tung University, Taipei, Taiwan, nctu.edu.tw; ^5^ School of Medicine, National Yang Ming Chiao Tung University, Taipei, Taiwan, nctu.edu.tw; ^6^ Department of Medical Research, Taipei Veteran General Hospital, Taipei, Taiwan

**Keywords:** electroencephalogram, general anesthesia, Holo-Hilbert spectral analysis, nociceptive–analgesic balance, Surgical Pleth Index

## Abstract

**Background:**

Intraoperative EEG provides a noninvasive window into cortical dynamics under anesthesia, but conventional spectral analysis cannot capture nonstationary modulation patterns linked to nociceptive processing. This study applied Holo‐Hilbert spectral analysis (HHSA) to characterize cross‐frequency modulation patterns in relation to the Surgical Pleth Index (SPI) during general anesthesia.

**Methods:**

Frontal EEG from 134 female patients undergoing gynecologic surgery was analyzed. Ten‐minute segments were first examined to define canonical modulation structures, followed by one‐minute epochs synchronized with SPI values to assess dynamic changes. HHSA decomposed each epoch into amplitude modulation patterns across carrier frequencies (1/64–64 Hz). Group comparisons between pain and no‐pain epochs were performed using *t*‐tests with Bonferroni correction. A linear mixed‐effects model evaluated the effects of SPI, minimum alveolar concentration (MAC), heart rate (HR), and mean arterial pressure (NIBP‐m) on alpha‐band modulation (8–16‐Hz carrier modulated by 3–8‐Hz amplitude).

**Results:**

HHSA revealed two dominant cross‐frequency interactions within the alpha‐carrier band (8–16 Hz): one modulated by 3–6‐Hz (high‐delta to theta) and another by 1–2‐Hz (low‐delta) oscillations, indicating layered modulation under anesthesia. During nociceptive states (SPI > 60), modulation power increased in the alpha and high‐delta bands, while theta and low‐delta modulation weakened. Alpha‐band modulation power rose with SPI and declined with MAC.

**Conclusions:**

HHSA revealed distinct cross‐frequency modulation patterns reflecting the cortical balance between nociception and analgesia. Alpha‐band modulation serves as a physiologically grounded EEG marker for individualized nociception monitoring under general anesthesia.

## 1. Introduction

In general anesthesia, analgesia has consistently been a critical component, alongside hypnosis, akinesia, and amnesia. It is employed not only to mitigate hemodynamic changes caused by surgical stimuli or to prevent body movement but also to reduce the surgical stress response, which involves both neuroendocrine–metabolic and inflammatory–immune components [1, 2]. Inadequate analgesia may lead to a more intense stress response, potentially resulting in negative short‐ and long‐term postoperative outcomes [3–5]. However, determining what constitutes “adequate analgesia” in unconscious anesthetized patients has traditionally been a challenging issue.

A previous study demonstrated that even in the absence of observable clinical responses to painful stimuli, spinal and brain neuronal activity was consistently present under general anesthesia [6]. The activation of functional magnetic resonance imaging signal in response to intense noxious stimuli gradually decreased with increasing remifentanil concentration, even beyond the level required to inhibit clinical responses. This finding suggests that commonly accepted clinical indicators may not be sufficient to accurately assess antinociception during general anesthesia. To address this, various nociception monitors, such as the Surgical Pleth Index (SPI), the Analgesia Nociception Index (ANI), and the Pupillary Pain Index (PPI), have been developed to better reflect the nociceptive–analgesic balance [4, 7, 8]. These monitors are primarily linked to autonomic nervous system activity and have been found to correlate with stress hormone levels. Although they have contributed to clinical anesthesia practice, they may be influenced by medications or other hemodynamic factors, complicating their interpretation [9].

Given that the brain is the primary target organ for general anesthesia, greater emphasis should be placed on understanding the effects of noxious stimuli on electroencephalogram (EEG) activity. A review by Paul S. García et al. highlighted that beta arousal, delta arousal, and alpha dropout are potential EEG characteristics observed in anesthetized patients under noxious stimuli [10]. Additionally, Wang et al. showed that, in addition to alpha dropout, the phase–amplitude modulation (AM) index of delta–alpha coupling decreases during painful stimulation and recovers after rescue opioids are administered [11]. Although these results are encouraging, the studies relied on conventional spectral‐based methods, such as Fourier transformation, where frequency bins of different bands are predefined before band‐pass filtering, limiting the detection of nonlinear interactions across different oscillatory components.

To comprehensively evaluate the nonlinear interactions between different components in nonstationary EEGs, we employed Holo‐Hilbert spectral analysis (HHSA) [12–14]. This method, an extension of the Hilbert–Huang transform (HHT) [15], provides detailed AM information for each carrier frequency band. We aim to use this adaptive technique to better understand EEG modulation patterns under general anesthesia and how these patterns vary with different nociceptive–analgesic balances, which may contribute to advancements in future anesthesia care.

## 2. Methods

### 2.1. Participants

This retrospective observational study was conducted at Chi Mei Medical Center in Tainan, Taiwan, between March 30, 2018, and June 30, 2020. The study protocol was approved by the Institutional Review Board of Chi Mei Medical Center (IRB serial number: 10703‐005), adhering strictly to the applicable STROBE criteria.

**Figure 1:**
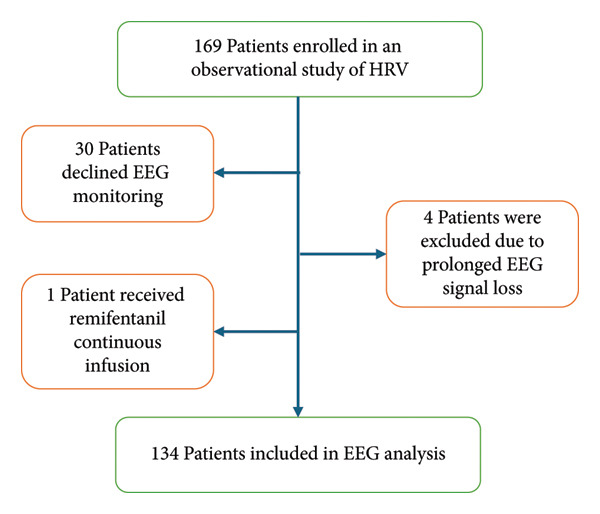
Flowchart of patient enrollment. HRV: Heart rate variability.

We used EEG data from a previous observational study on heart rate variability (HRV) during gynecological surgery that initially enrolled 169 female patients aged 20–80 years (ASA Physical Status I–III). Of these, 134 patients were included in the current EEG analysis. Thirty‐five patients were excluded from the analysis: 30 did not consent to EEG monitoring (only HRV monitoring was performed), four experienced prolonged intraoperative EEG signal loss (greater than 20 min) due to equipment malfunction, and 1 received continuous remifentanil infusion instead of the standard bolus fentanyl‐morphine regimen, making it impossible to compare analgesic dosages (Figure 1).

### 2.2. Anesthesia Techniques

There was no administration of medicine before the surgery. Every patient was observed using a three‐lead ECG, plethysmography, a noninvasive blood pressure monitor, a GE (GE Healthcare Finland Oy, Helsinki, Finland) neuromuscular blockade monitor, a GE entropy sensor for a brain wave–derived anesthesia depth monitor, and other monitors recommended by the ASA as standard for basic anesthesia monitoring in the operating room. The induction drugs used were fentanyl at a dose of 1.5–2 μg kg^−1^, xylocaine at a dose of 0.5–1 mg kg^−1^, propofol at a dose of 1.5–2 mg kg^−1^, and cisatracurium at a dose of 0.15–0.2 mg kg^−1^. Every patient was administered balanced general anesthesia using halogenated anesthetic drugs. The desired range for the state entropy (SE) level during the operation was set at 40 to 60. The SPI was employed to assess the equilibrium between nociceptive and antinociceptive responses during anesthesia. Prior to being transferred to the postanesthesia care unit (PACU), all patients were extubated.

### 2.3. Data Acquisition and Preprocessing

Physiological signals were continuously recorded from a GE Datex‐Ohmeda S/5 Anesthesia Monitor and streamed to a dedicated computer using the S/5 iCollect system (GE Healthcare). Frontal EEG for entropy monitoring was obtained with the GE entropy module using a bifrontal two‐lead montage (Fp1–Fp2) referenced according to the manufacturer’s layout. This frontal montage was selected because anesthetic‐induced alpha oscillations reliably shift toward anterior regions during unconsciousness, making frontal EEG the most sensitive site for capturing these dynamics [16–18]. In addition, frontal placement is the standard configuration for intraoperative depth‐of‐anesthesia monitors and allows rapid, unobtrusive application in the surgical field. Electrode impedances were maintained below 5 kΩ throughout the recording. EEG signals were digitized at a sampling rate of 300 Hz and band‐pass filtered between 0.1 and 64 Hz using the built‐in acquisition filters.

Continuous EEG recordings were visually inspected by an anesthesiologist experienced in intraoperative EEG interpretation. For each candidate 10‐min segment, periods showing amplifier saturation, electrode detachment, or gross artifacts (e.g., sustained movement or surgical cautery) were quantified. Segments containing more than 2 min of such artifacts were discarded entirely to avoid introducing discontinuities or bias from partial signal removal. Segments with ≤ 2 min of artifact were retained without further excision and were processed in their entirety for HHSA.

### 2.4. HHSA Preprocessing and Parameter Settings

EEG signals were *z*‐scored and decomposed using the adaptive Holo‐ACE algorithm implemented via RCADA‐AEEMD. Each 10‐min artifact‐free segment was analyzed with 10 ensemble realizations and five noise levels (0.04–0.20, step 0.04), using quadratic sifting as the stopping criterion. The number of intrinsic mode functions (IMFs) was automatically determined according to the signal length.

To characterize minute‐by‐minute spectral dynamics, EEG recordings were further divided into 1‐min epochs and decomposed with Holo‐ACE using zero‐crossing sifting, 10 ensembles, and up to seven IMFs (nimf = 7) at a fixed noise level of 0.2. The fixed noise amplitude ensured consistent decomposition across more than 13,000 epochs while substantially reducing computational load. Carrier (FM) and modulation (AM) frequencies were represented on an 80 × 80 logarithmic grid covering FM = 1/64–64 Hz and AM = 1/32–32 Hz. For visualization, spectra were displayed within FM = 1/16–64 Hz and AM = 1/8–32 Hz to enhance readability.

### 2.5. HHSA

To analyze the frequency modulation and AM of EEG signals in this study, we employed HHSA, a technique advanced by Norden E. Huang in 2016 [12]. Unlike traditional spectral analysis methods such as Fourier and wavelet transforms, which are constrained by assumptions of linearity and stationarity, HHSA offers a more sophisticated approach by leveraging a hierarchical decomposition process. Given that EEG signals recorded under general anesthesia are inherently nonstationary and exhibit pronounced nonlinear dynamics, conventional methods can obscure or inaccurately estimate the true instantaneous frequency modulation and AM. HHSA overcomes these limitations by leveraging a hierarchical decomposition process, facilitating a multilevel decomposition of EEG signals into IMFs. This framework enables detailed characterization of AM and frequency modulation across multiple scales, providing a more comprehensive spectral representation of anesthetized EEG activity.

One key advantage of HHSA is its ability to capture nonlinear interactions embedded within the signal. In the present study, the term cross‐frequency coupling (CFC) refers specifically to interactions between oscillatory components at different frequencies within the same EEG channel, as reflected by the hierarchical amplitude–frequency modulation patterns extracted through HHSA. This usage denotes spectral–temporal relationships within a single frontal recording and does not imply connectivity or coupling between spatially distinct cortical regions.

HHSA was applied to perioperative EEG data to extract detailed time–frequency–amplitude representations. The resulting spectra, derived from hierarchical decomposition, were analyzed to identify specific patterns of neural oscillations and AM structures that may provide insight into neural states and anesthetic responsiveness [19].

### 2.6. Primary Outcome and Secondary Outcome

The primary outcome of this study was to characterize the canonical modulation pattern of the EEG during general anesthesia. To minimize transient fluctuations and emphasize stable oscillatory behavior, 10‐min artifact‐free epochs were analyzed to identify consistent cross‐frequency modulation structures. These epochs were restricted to the intermediate nociceptive range (SPI = 40–60), representing a typical balance between nociception and analgesia during surgical maintenance. The resulting pattern of carrier‐frequency activity modulated by lower‐frequency amplitude oscillations served as the reference framework for subsequent comparisons.

The secondary outcome examined differences in modulation patterns under varying nociceptive conditions, defined as pain (SPI > 60) and no‐pain (SPI < 40). To capture temporal dynamics of the nociceptive–analgesic balance, EEG recordings were segmented into 1‐minute epochs and classified according to the corresponding SPI values. For quantitative analysis, modulation power was extracted from a physiologically meaningful region corresponding to alpha‐band modulation, defined as carrier frequency (FM) 8–16 Hz modulated by amplitude frequency (AM) 3–8 Hz. This region showed the largest pain–no‐pain contrast and was used in subsequent statistical modeling to examine its relationship with nociceptive and anesthetic variables.

### 2.7. Statistical Analysis

The statistical analysis aimed to compare EEG modulation patterns between pain (SPI > 60) and no‐pain (SPI < 40) groups using the results from HHSA. For each one‐minute EEG segment, HHSA produced an 80 × 80 matrix, with carrier frequencies (columns) and modulation frequencies (rows) distributed on a logarithmic scale.

Group‐level comparisons were first conducted using unequal‐variance two‐sample *t*‐tests at each point of the matrix to identify cross‐frequency differences between the pain and no‐pain groups. To control for multiple comparisons, the Bonferroni correction was applied to maintain a family‐wise significance level of *p* < 0.05.

To account for within‐subject variability, a paired *t*‐test was subsequently performed to compare modulation patterns between pain and no‐pain epochs for individual patients. A Bonferroni correction was also applied to the results of this analysis to maintain statistical rigor and mitigate potential confounding factors.

For secondary regression analyses, a linear mixed‐effects model (LME) was used to examine whether the association between SPI and alpha‐band modulation remained significant after adjusting for anesthetic depth and hemodynamic covariates. Competing random‐intercept and random‐slope structures were compared via likelihood‐ratio tests, and model assumptions were verified through residual normality and homoscedasticity diagnostics.

## 3. Results

### 3.1. Demographics and Perioperative Conditions of Patients

The average age of the patients included in the study was 44.4 years. Fifty‐nine percent of them underwent laparoscopic surgery, while 41% had open surgeries. The average duration of surgery was 134.6 min, with an average blood loss of 165.6 mL. The opioids administered during surgery were limited to fentanyl and morphine, with average doses of 114.2 μg and 4.8 mg, respectively. The initial pain intensity after the arrival of the postoperative care unit, measured on the numeric rating scale, was 3.0 on average (Table 1).

**Table 1:** Clinical and demographic characteristics.

Measure		Participants, *n* = 134	
Age (years)		44.4 ± 10.3	
Body mass index (kg/m^2^)		23.3 ± 3.7	
Blood loss (mL)		165.7 ± 205.7	
Fentanyl dosage (μg)		114.2 ± 40.5	
Morphine dosage (mg)		4.8 ± 2.2	
Surgery time (min)		134.6 ± 45.0	
First NRS score in POR unit		3.0 ± 2.2	

		**Number**	**Percentage (%)**

ASA physical status	I	18	13.4
	II	98	73.1
	III	18	13.4
Surgical type	Open	55	41
	Laparoscope	79	59

*Note:* Values are mean (SD) or number (proportion).

### 3.2. Methodological Grounding of HHSA

To establish the fundamental difference between the nonlinear analysis utilized in this study and conventional spectral methods, a side‐by‐side comparison of the HHT and a traditional linear spectral estimate is provided (Figure 2). This comparison utilizes an identical five‐minute EEG segment recorded under general anesthesia (SPI between 40 and 60). Panel A displays the conventional linear time–frequency estimate, specifically the density spectral array (DSA) derived from a short‐time Fourier transform, using a 2‐s Hamming window with 90% overlap across the 0.2–30‐Hz band. Panel B presents the nonlinear Hilbert–Huang time–frequency representation of the same 0.2–30‐Hz EEG segment, computed after empirical mode decomposition. The results visually demonstrate that the nonlinear instantaneous frequency–based representation is essential for capturing the complex, nonstationary dynamics characteristic of EEG under anesthesia. This methodological validation justifies the subsequent use of the HHSA framework for our primary analyses.

Figure 2Comparison between a conventional linear spectral estimate and the nonlinear Hilbert–Huang transform (HHT) for the same five‐minute EEG segment recorded under general anesthesia during stable depth, with the Surgical Pleth Index (SPI) and state entropy (SE) both maintained between 40 and 60. Panel A shows the density spectral array (DSA) derived from a short‐time Fourier transform using a 2‐s Hamming window with 90% overlap across the 0.2–30‐Hz band, with power displayed in decibels. Panel B presents the Hilbert–Huang time–frequency representation of the same 0.2–30 Hz EEG segment, computed after empirical mode decomposition and visualized with low‐frequency masking below 0.24 Hz for clarity. Together, these panels provide a side‐by‐side illustration of the differences between traditional linear spectral estimation and the nonlinear instantaneous frequency–based representation used in our HHSAs.

**Figure:**
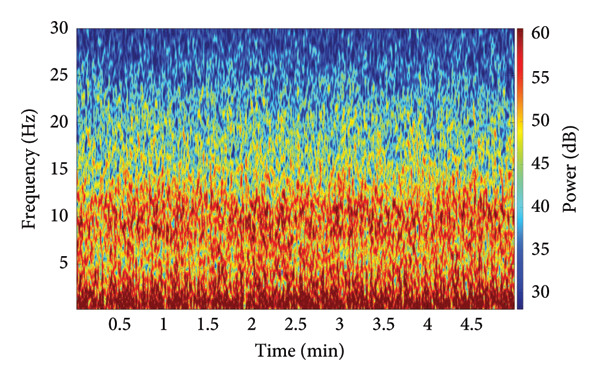
(a)

**Figure:**
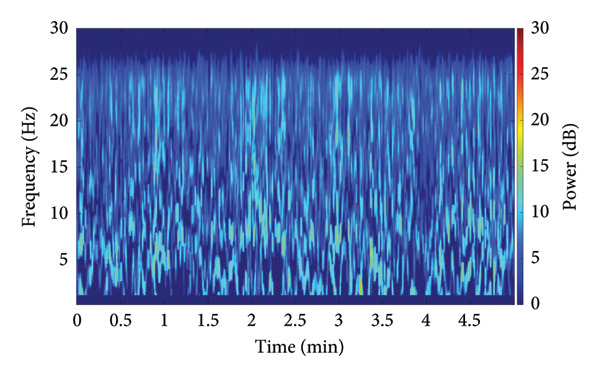
(b)

### 3.3. Primary Outcome: Common Modulation Patterns of EEGs Under General Anesthesia on HHSA

The HHSA method is proficient in managing intramode and intermode nonlinear modulations in both stationary and nonstationary processes. Consequently, we employed this method to unveil the complex nonlinear interactions within EEGs during anesthesia.

**Figure 3:**
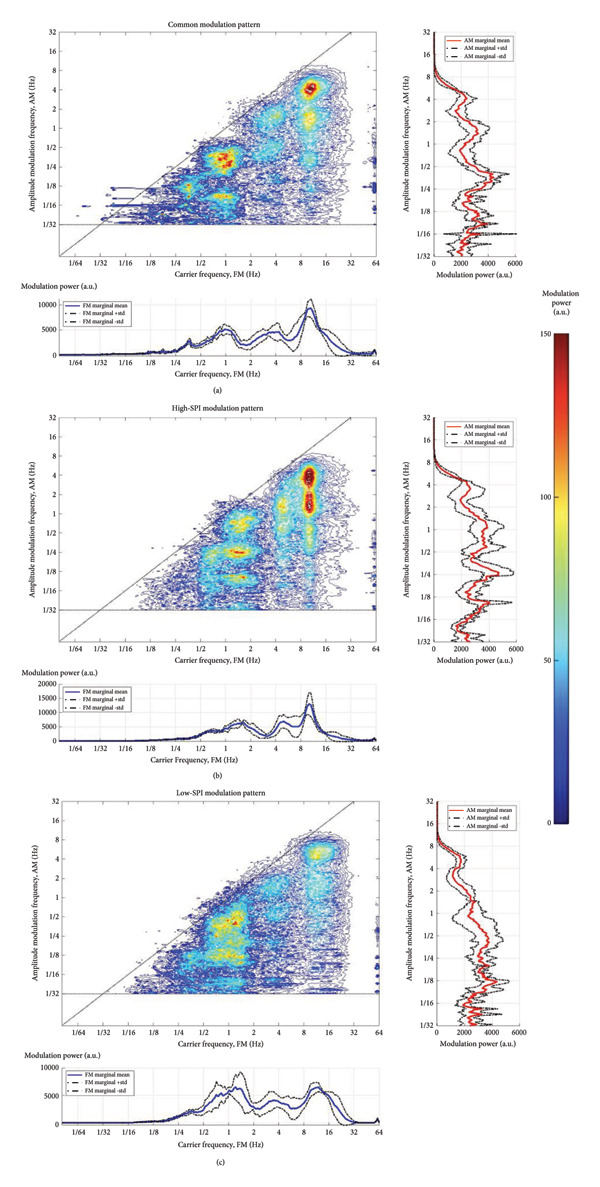
(a) Common EEG modulation patterns during general anesthesia processed by Holo‐Hilbert spectral analysis (HHSA). This panel presents the results from a 10‐min EEG recording of a healthy woman in her forties, undergoing general anesthesia for a gynecological operation. The depth of anesthesia was maintained within a range of 40–60, as measured by an entropy monitor. Concurrently, the nociceptive–analgesic balance was kept between 40 and 60, monitored through the Surgical Pleth Index (SPI). Throughout the data acquisition, her vital signs remained stable, illustrating typical physiological conditions under general anesthesia during surgery. (b) EEG modulation patterns processed by Holo‐Hilbert spectral analysis (HHSA) under conditions with higher analgesic stimulus. This panel shows the EEG modulation patterns when the Surgical Pleth Index (SPI) is greater than 60, while the depth of anesthesia remains between 40 and 60. (c) EEG modulation patterns processed by Holo‐Hilbert spectral analysis (HHSA) under conditions with lower analgesic stimulus. This panel illustrates the EEG modulation patterns when the Surgical Pleth Index (SPI) is less than 40, while the depth of anesthesia remains between 40 and 60.

Our HHSA of EEG data reveals detailed modulation patterns as patients reach an anesthesia depth suitable for surgery (Figure 3). Notably, the alpha band (8–16 Hz) modulated by the oscillation at 3–6 Hz (high‐delta to theta band) displayed the most substantial energy distribution, indicating a prominent interaction at these frequencies. The alpha‐band oscillations were also modulated by the low‐delta band (1–2 Hz) and other lower‐frequency components, demonstrating the complexity of brain activity under anesthesia.

While the oscillations around 3–6 Hz were similarly influenced by the low‐delta band (1–2 Hz) and slower cycles, these interactions resulted in considerably lower energy levels compared to the alpha‐band oscillations. Moreover, frequencies below 2 Hz showed elevated energy densities; however, their distribution was notably irregular, highlighting the chaotic nature of anesthetized brain states.

Modulations of high‐delta and low‐delta oscillations on the carrier alpha wave and modulations of slow waves on the carrier low‐delta wave were consistently observed across 10‐min epochs from different individuals. Nevertheless, the specific energy distributions varied under different conditions.

### 3.4. Secondary Outcome

#### 3.4.1. Visualization of Cortical and Autonomic Dynamics Across Time

To provide a holistic physiological context for the HHSA findings and address the nonstationary nature of the data, Figure 4 illustrates the temporal correspondence between EEG modulation patterns and clinical nociceptive indices over a representative 9‐min epoch. Panel A displays the time course of aggregate modulation power within the delta (1–4 Hz) and alpha (8–16 Hz) carrier‐frequency bands, which correspond to the principal modulation foci identified in our primary results. Panel B shows the simultaneous fluctuations in the SPI, reflecting autonomic nociceptive balance, and the end‐tidal minimum alveolar concentration (MAC), reflecting anesthetic depth. Panel C presents the underlying HHT spectrum (0.1–30 Hz) for complementary visualization of spectral dynamics. Panel D further summarizes the evolution of AM power across time by projecting the HHSA output onto the AM‐frequency axis (≤ 10 Hz). This representation highlights slow‐frequency modulation components that exhibited significant effects in our statistical analyses and provides a time‐resolved view of modulation amplitude that is not captured by conventional spectral plots.

Figure 4Temporal visualization of Holo‐Hilbert spectral analysis (HHSA) metrics synchronized with clinical parameters over a 9‐min epoch. Panel A illustrates the aggregate modulation power within the delta (1–4 Hz) and alpha (8–16 Hz) carrier‐frequency bands. The left *y*‐axis (delta‐band modulation) is scaled in arbitrary units of 10^4^ (a.u.), and while the right *y*‐axis (alpha‐band modulation) displays absolute power values (a.u.). Panel B depicts the simultaneous fluctuations in the Surgical Pleth Index (SPI), reflecting nociceptive balance, and the end‐tidal minimum alveolar concentration (MAC), reflecting anesthetic depth. Panel C provides the Hilbert–Huang transform (HHT) time–frequency spectrum (0.1–30 Hz), offering complementary visualization of instantaneous spectral dynamics. Panel D visualizes the evolution of amplitude–modulation (AM) power over time by projecting the HHSA output onto the AM‐frequency axis (≤ 10 Hz). For each 1‐min EEG epoch, the full FM dimension was collapsed to yield a two‐dimensional representation of AM power. Warmer colors indicate stronger modulation power. This panel highlights gradual shifts in low‐frequency AM components that are not apparent in conventional spectral or instantaneous frequency displays and complements Panels A–D by demonstrating how slow‐frequency modulation power varies in parallel with changes in nociceptive and autonomic states.

**Figure:**
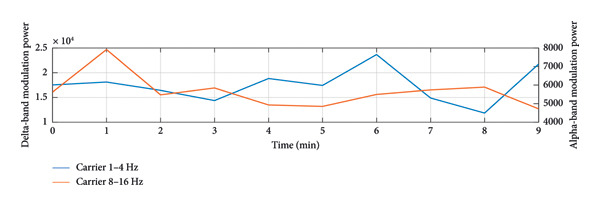
(a)

**Figure:**
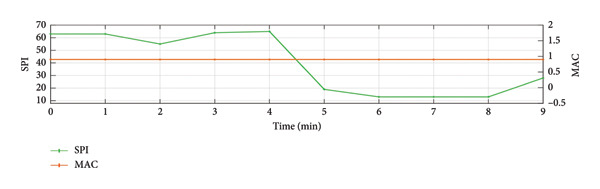
(b)

**Figure:**
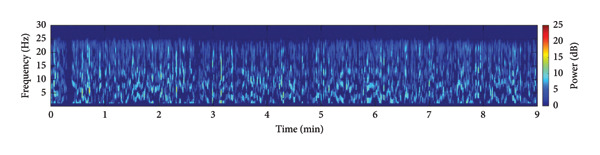
(c)

**Figure:**
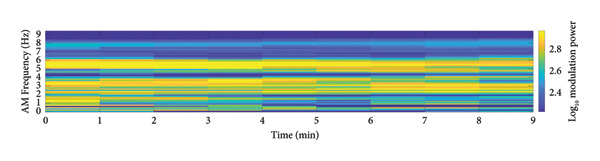
(d)

Qualitatively, the parallel evolution of HHSA‐derived carrier‐band modulation power and SPI demonstrates a strong temporal link between cortical dynamics and autonomic nociceptive responses within the same recording window. The relative stability of the MAC trace throughout the epoch further supports the interpretation that fluctuations in EEG modulation power primarily reflect the nociceptive–antinociceptive balance, rather than changes in anesthetic administration.

#### 3.4.2. Differences in Energy Distribution During Epochs of Different Nociceptive–Analgesic Balance Status

The EEG recordings obtained during general anesthesia were systematically divided into 1‐minute segments. These segments were then classified according to the SPI values associated with each epoch. Segments where the SPI exceeded 60 were designated as the pain group, indicating a higher level of nociceptive activity. Conversely, segments where the SPI was under 40 were categorized as the no‐pain group, reflecting a lower nociceptive state.

Each EEG segment was preprocessed to ensure data quality and consistency prior to analysis with the HHSA. The HHSA results for each segment were visualized in an 80 × 80 matrix, where each element represents a specific point on the plane defined by the carrier frequency (*x*‐axis) and the frequency of AM (*y*‐axis). Higher values in this matrix denote higher energy levels at the corresponding frequency interactions, indicating more intense modulations.

**Figure 5:**
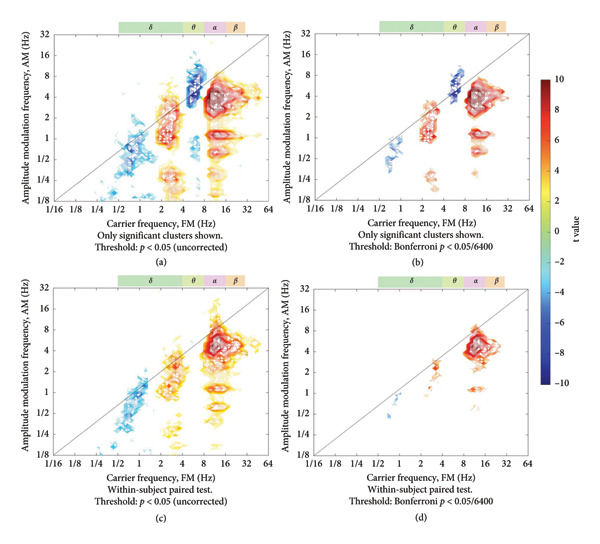
Statistical analysis of EEG data during painful and nopain epochs. This figure illustrates the t‐stats between EEG recordings during episodes with a high Surgical Pleth Index (SPI > 60, indicating pain) and those with a low SPI (< 40, indicating no pain). (a) Results from an independent *t*‐test show significant differences in modulation power, with higher values in the high‐delta (2–4 Hz), alpha (8–16 Hz), and beta bands during painful epochs, and lower values in the theta band (4–8 Hz), lower‐delta (1–2 Hz), and slow‐wave (< 1 Hz) regions. (b) Application of the Bonferroni correction confirms the robustness of these findings. (c) A paired *t*‐test comparing painful and nonpainful epochs largely corroborates the results from (a), although the differences in the alpha band are less pronounced. (d) Results from a paired *t*‐test after applying the Bonferroni correction to account for multiple comparisons. The color scale represents the t‐statistic value, where red areas (*t* > 0) indicate significantly higher modulation power in the pain group (SPI > 60), and blue areas (*t* < 0) indicate significantly lower power.

To compare the pain and no‐pain groups, we conducted an independent *t*‐test for each of the 6400 points in the HHSA matrix. The points exhibiting statistically significant differences were plotted along with their *t*‐statistics in Figure 5(a). This visualization highlights areas where frequency modulation patterns significantly differ between conditions. Notably, the most pronounced differences occur at the intersection of the carrier‐frequency range of 8–16 Hz with the AM frequency range of 3–8 Hz. In these regions, the energy distribution was significantly higher during pain epochs compared to the no‐pain epochs. Interestingly, in the area of the carrier frequency of 4–8 Hz, the modulation by the frequency of 3–4 Hz was significantly weaker in the pain epochs compared to the no‐pain epochs, while the opposite was observed in the area of the carrier frequency 2–4 Hz. For carrier frequencies below 2 Hz, the energy distribution was weaker in the pain epochs.

To adjust for multiple comparisons, we performed a Bonferroni correction with the alpha value set to 0.05 divided by 6400. The results are illustrated in Figure 5(b). The differences occurring at the intersection of the carrier‐frequency range of 8–16 Hz with the AM frequency range of 3–8 Hz remained robust. Additionally, the weaker modulation in the section of 4–8 Hz and the stronger modulation in the section of 2–4 Hz could still be observed in the pain epochs.

We also compared epochs with pain and epochs without pain within the same subject. The results of the intrasubject analysis are shown in Figure 5(c), and the results of the Bonferroni correction are depicted in Figure 5(d). The patterns observed were similar to those shown in Figures 5(a) and 5(b), except for the subtle variations in the modulation of the carrier‐frequency range of 4–8 Hz, which were not present in the intrasubject analysis.

#### 3.4.3. Subanalysis of Each Grid on the Plane of Carrier Frequency and AM Frequency

To facilitate comparison, we categorized the frequency range in our study as follows: 0.5–1 Hz as slow waves, 1–2 Hz as low‐delta waves, 2–4 Hz as high‐delta waves, 4–8 Hz as theta waves, 8–16 Hz as alpha waves, and 16–30 Hz as beta waves. Each grid represents the intersection of a specific range of carrier frequency and a specific range of AM frequency. We calculated the two‐dimensional summation of each grid and compared the total energy in each grid between pain epochs and no‐pain epochs. Notably, only intermodal modulations (modulations across scales where the carrier frequency is greater than the AM frequency) are presented in Table 2.

**Table 2:** Comparative analysis of EEG modulation power during painful and non‐painful epochs.

**Brainwave modulation power (pain epochs vs. nopain epochs = 3348:10227)**																
		**Amplitude Modulation**														
		**0.5–1 Hz**			**1-2 Hz**			**2–4 Hz**			**4–8 Hz**			**8–16 Hz**		
		**Pain**	**Nopain**	***p* value**	**Pain**	**Nopain**	***p* value**	**Pain**	**Nopain**	***p* value**	**Pain**	**Nopain**	***p* value**	**Pain**	**Nopain**	***p* value**

Frequency Modulation	1‐2 Hz	17.0 ± 13.7	17.8 ± 14.4	0.004												
	2–4 Hz	15.4 ± 6.7	14.4 ± 6.4	< 0.001	28.1 ± 10.9	25.7 ± 12.4	< 0.001									
	4–8 Hz	6.5 ± 4.1	6.9 ± 4.5	< 0.001	10.3 ± 4.9	10.5 ± 5.4	0.04	9.2 ± 6.2	9.5 ± 6.9	0.04						
	8–16 Hz	18.4 ± 8.3	16.9 ± 8.4	< 0.001	24.2 ± 13.9	22.4 ± 13.8	< 0.001	23.8 ± 11.3	20.8 ± 11.0	< 0.001	30.5 ± 12.2	28.0 ± 12.6	< 0.001			
	16 –32 Hz	3.9 ± 1.9	3.7 ± 1.9	< 0.001	5.1 ± 3.3	4.8 ± 3.1	< 0.001	3.0 ± 1.4	2.7 ± 1.4	< 0.001	5.4 ± 3.2	5.0 ± 2.9	< 0.001	1.1 ± 1.0	1.1 ± 1.0	0.35

In the low‐delta band, the modulation of the carrier wave by the slow wave was significantly weaker during the pain epochs. Conversely, in the high‐delta band, the carrier wave was modulated more during the pain epochs, by either the low‐delta wave or the slow wave. The condition changed again in the theta band, where the energy of the amplitude frequency modulation was lower across the slow wave, low‐delta wave, and high‐delta wave. The results flipped again when examining the alpha‐carrier wave, which showed stronger modulation in the frequency range from the slow wave to the theta wave. The beta carrier wave exhibited a similar pattern of modulation as the alpha wave, with higher energy distribution in the pain group. Interestingly, the AM by the alpha wave did not differ between the groups.

#### 3.4.4. Mixed‐Effects Model of Alpha‐Band Modulation

Based on the dichotomous comparison between pain (SPI > 60) and no‐pain (SPI < 40) epochs, the region showing the most prominent difference in modulation power was located at carrier frequencies between 8 and 16 Hz and AM frequencies between 3 and 8 Hz, corresponding to the alpha‐band activity modulated by theta and upper delta range amplitude fluctuations. This region was therefore selected as the target for subsequent regression analysis to quantify how alpha‐band modulation varies with nociceptive and anesthetic factors at the per‐epoch level.

A LME was then fitted with the modulation power within this region as the dependent variable and the SPI, age‐adjusted MAC fraction, SE, mean arterial pressure (NIBP‐m), and heart rate (HR) as fixed effects. Random intercepts and slopes for SPI were included to capture interindividual variability in baseline EEG dynamics and nociceptive sensitivity. Epochs with SE > 70 or MAC < 0.5 were excluded to ensure stable anesthetic depth.

The final model demonstrated a significant positive association between SPI and alpha‐band modulation (*β* = 4.3 ± 1.1, *p* < 0.001), indicating that stronger nociceptive stimulation was accompanied by increased modulation power in the alpha‐carrier band. In contrast, higher MAC values were associated with a significant suppression of modulation power (*β* = −874.6 ± 46.4, *p* < 0.001), HR exhibited a modest but significant negative effect (*β* = −2.2 ± 0.6, *p* < 0.001), whereas NIBP‐m showed no significant association (*p* = 0.21). The likelihood‐ratio test comparing random‐intercept and random‐slope structures favored the latter, indicating that allowing patient‐specific slopes for SPI improved model fit and captured substantial interindividual variability in alpha‐band modulation sensitivity.

**Figure 6:**
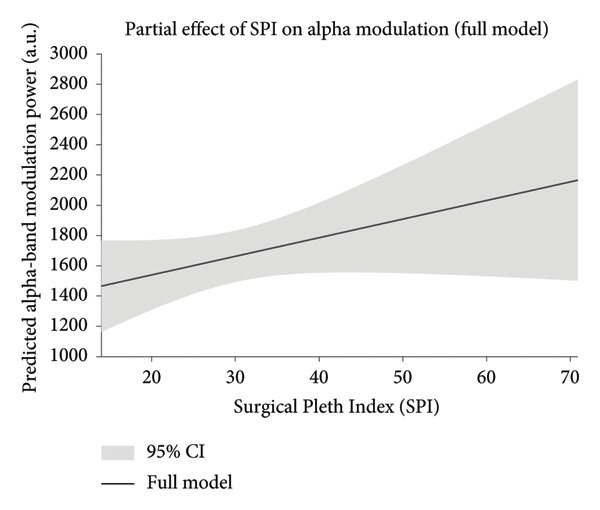
Partial effect of the Surgical Pleth Index (SPI) on alpha‐band modulation after adjustment for anesthetic and hemodynamic covariates: Predicted relationship between SPI and modulation power in the alpha‐carrier band, based on the linear mixed‐effects model that included age‐adjusted MAC fraction, state entropy (SE), mean arterial pressure (NIBP‐m), and heart rate (HR) as covariates. The dark red line represents the model‐predicted effect of SPI, and the light blue shaded area indicates the 95% confidence interval. Each prediction was generated while holding all other variables at their median values. The analysis was restricted to epochs with SE ≤ 70 and MAC ≥ 0.5 to ensure stable anesthetic depth.

Model diagnostics confirmed acceptable residual normality and homoscedasticity. The partial‐effect plot (Figure 6) illustrates the predicted relationship between SPI and alpha‐band modulation after controlling for anesthetic and hemodynamic covariates, with the 95% confidence interval shown as a semitransparent shaded area. Detailed model coefficients are summarized in Tables 3 and 4.

**Table 3:** Fixed‐effects coefficients from the linear mixed‐effects model.

Predictor	Coefficient estimate (*β*)	Standard error (SE)	*t* statistic	*p* value	95% confidence interval (CI)
Intercept	3193.4	95.4	33.5	< 0.001^∗∗∗^	[3006.5, 3380.4]
SPI	4.3	1.1	4.0	< 0.001^∗∗∗^	[2.2, 6.4]
HR	−2.2	0.6	−3.7	< 0.001^∗∗∗^	[−3.4, −1.1]
NIBP_mean_	0.6	0.5	1.3	0.209	[−0.32, 1.46]
MAC	−874.6	46.4	−18.0	< 0.001^∗∗∗^	[−965.5, −783.6]

*Note:* The model formula is as follows: Alpha_mod∼1 + SPI + HR + NIBP_m + MAC + (1 + SPI∣Patient).

*p* values: ^∗^ *p* < 0.05; ^∗∗^ *p* < 0.01; ^∗∗∗^ *p* < 0.001.

**Table 4:** Random‐effects covariance parameters.

Random effect component	Type	Estimate	95% confidence interval (CI)
Group: patient (levels = 136)			
Standard deviation of intercept (*σ* intercept)	Std	853.7	[753.41, 967.41]
Standard deviation of SPI slope (*σ* SPI)	Std	11.4	[9.84, 13.09]
Correlation between intercept and SPI slope (*ρ* intercept, SPI)	Corr	−0.4	[−0.54, −0.23]

**Residual**			

Residual standard deviation (*σ* error)	Residual Std	599.3	[593.0, 605.7]

Abbreviations: corr, correlation; Std, standard deviation.

## 4. Discussion

The present study revealed that EEG under general anesthesia exhibited prominent modulation foci within the alpha (8–16 Hz) and low‐delta (1–2 Hz) carrier‐frequency ranges. Among these, alpha‐band modulation—particularly the 8–16‐Hz carrier modulated by 3–8‐Hz amplitude fluctuations—showed the strongest sensitivity to nociceptive intensity. Modulation power increased with higher SPI values, while decreasing with higher anesthetic concentrations, suggesting that alpha modulation reflects cortical responsiveness to nociceptive input independent of anesthetic depth.

Previous studies have described several EEG responses to noxious stimulation, including beta arousal, delta arousal, and alpha dropout [10, 11, 20]. Consistent with these reports, our analysis identified enhanced modulation within the high‐delta (2–4 Hz) and reduced modulation within the low‐delta (1–2 Hz) bands during intense nociceptive periods. Although the physiological basis remains uncertain, delta activity has repeatedly been linked to nociceptive processing even under anesthesia [21], and similar low‐frequency changes (< 2 Hz) have been reported in ICU patients [20].

Our analysis revealed that the most pronounced reduction in modulation power between pain (SPI > 60) and no‐pain (SPI < 40) epochs occurred within the 4–8‐Hz carrier‐frequency band, differing from the “alpha dropout” commonly described in prior literature [10, 11, 22]. The theta oscillations modulated by slower delta activity were markedly attenuated under stronger nociceptive conditions. One plausible explanation for this discrepancy lies in the inconsistent definitions of the alpha band across studies. Reported ranges vary widely—from 8–12 Hz to 8–14 Hz, or even 6–17 Hz [10, 11, 23]—reflecting the fact that most analyses focused on canonical EEG bands linked to distinct neural generators, such as thalamocortical oscillations, rather than on data‐driven spectral structures. Consequently, our findings may represent a broader manifestation of “alpha dropout,” extending into the theta range when examined through adaptive spectral decomposition.

This discrepancy may also reflect methodological differences. Unlike traditional Fourier‐ or wavelet‐based approaches that use fixed frequency bins, the EEMD‐based HHSA framework adaptively decomposes EEG signals into IMFs based on local oscillatory features. Conventional DSA displays power that has been averaged through fixed windows and basis functions, which inevitably smear energy across neighboring frequencies and produce broader, more diffuse color patterns. Wavelet representations offer better temporal resolution but remain convolution‐based, and therefore, their energy distribution still reflects the width of the wavelet kernel rather than the true oscillatory trajectory. In contrast, the HHT yields narrow instantaneous‐frequency ridges with tightly localized amplitude because it derives frequency and amplitude directly from the analytic signal of each IMF. The HHSA further extends this framework by quantifying how the amplitude of one IMF is modulated by the instantaneous frequency of another, revealing a modulation structure that cannot be captured by DSA, wavelet, or the HHT alone. The observed attenuation of theta‐band modulation during high‐SPI epochs may therefore reflect a genuine alteration in CFC rather than a fixed‐band power change. Nevertheless, such variations were not uniformly observed across subjects, suggesting that individual traits or state‐dependent cortical dynamics unrelated to nociceptive–analgesic balance could also contribute to the heterogeneity.

The mixed‐effects model further demonstrated opposing effects of nociceptive and anesthetic factors on alpha‐band modulation (8–16 Hz modulated by 3–8 Hz), with SPI showing a positive and MAC showing a negative association with modulation power. This bidirectional relationship implies that nociceptive activation enhances cortical coupling within this frequency range, whereas volatile anesthetics suppress it in a dose‐dependent manner. Previous studies have reported transient beta activation (12–25 Hz) under lighter anesthesia when cortical networks remain partially responsive to noxious stimuli [10, 24, 25]. In our data, the region showing the largest modulation difference encompassed both the “traditional” alpha and lower beta bands, indicating that even at depths associated with adequate hypnosis (SE < 70), cortical dynamics retain residual sensitivity to nociceptive perturbations. The progressive increase in cross‐frequency modulation (8–16 Hz by 3–8 Hz) with higher SPI values likely reflects this remaining cortical reactivity to nociceptive input.

The observed modulation structure supports the interpretation that CFC reflects large‐scale functional coordination in the anesthetized brain. Previous studies have shown that delta–alpha coupling strength correlates with anesthetic depth and thalamocortical synchrony that maintains unconsciousness [26–28]. The two modulation foci identified in our 10‐min analysis (3–6 Hz and 1–2 Hz modulating 8–16 Hz) may therefore represent distinct pathways of thalamocortical and slower cortico‐subcortical interaction during anesthesia.

This study has several limitations. EEG data were acquired exclusively from frontal electrodes of an anesthesia monitor, limiting spatial interpretation of large‐scale network dynamics. All participants were female patients undergoing gynecological surgery, which may restrict generalizability to other populations or procedures. Additionally, the single‐center design and moderate sample size warrant validation in larger, more diverse cohorts.

In conclusion, this study highlights the capability of HHSA to uncover dynamic cross‐frequency EEG modulation patterns associated with nociceptive–analgesic balance under general anesthesia. Distinct AM foci within the alpha bands likely reflect separate thalamocortical and cortico‐subcortical pathways. The opposing effects of SPI and MAC on alpha‐band coupling suggest that cross‐frequency modulation provides a sensitive and physiologically grounded marker of cortical responsiveness, offering potential utility for individualized intraoperative monitoring.

## Ethics Statement

The study was conducted in accordance with the Declaration of Helsinki, and the protocol was approved by the Institutional Review Board of Chi Mei Medical Center (IRB serial number: 10703‐005).

## Disclosure

We confirm that no individuals or third‐party services contributed to the study or manuscript preparation other than those named as authors in the manuscript.

## Conflicts of Interest

The authors declare no conflicts of interest.

## Author Contributions

Chun‐Ning Ho: conceptualization, methodology, formal analysis, writing–original draft preparation. Norden E. Huang: software, supervision. Jen‐Yin Chen: conceptualization, resources, Albert C. Yang: writing–review and editing, supervision, funding acquisition.

## Funding

This work was supported by grants from the National Science and Technology Council, Taiwan (grant numbers NSCT 114‐2321‐B‐A49‐011, 114‐2321‐B‐A49‐003, 113‐2634‐F‐A49 ‐003). Albert C. Yang was also supported by the Mt. Jade Young Scholarship Award from the Ministry of Education, Taiwan, as well as the Brain Research Center, National Yang Ming Chiao Tung University, and the Ministry of Education (Aim for the Top University Plan), Taipei, Taiwan.

## Data Availability

The EEG datasets generated and analyzed during the current study are not publicly available due to privacy and ethical restrictions but are available from the corresponding author upon reasonable request.
